# Hemopneumothorax detection through the process of artificial evolution - a feasibility study

**DOI:** 10.1186/s40779-021-00319-2

**Published:** 2021-04-25

**Authors:** Adir Sommer, Noy Mark, Gavriel D. Kohlberg, Rafi Gerasi, Linn Wagnert Avraham, Ruth Fan-Marko, Arik Eisenkraft, Dean Nachman

**Affiliations:** 1 The Medical Corps, Israel Defense Forces (IDF), Tel Hashomer, 5262000 Ramat Gan, Israel; 2 Department of Otolaryngology - Head and Neck Surgery, University of Washington, Seattle, WA 98195 USA; 3 The Institute for Research in Military Medicine, the Faculty of Medicine, the Hebrew University of Jerusalem and the IDF Medical Corps, 9112102 Jerusalem, Israel; 4 The Hebrew University School of Medicine, 9112102 Jerusalem, Israel; 5 Department of Medicine, Hadassah Medical Center, 9112102 Jerusalem, Israel

**Keywords:** Pneumothorax, Hemothorax, Trauma, Battlefield, Artificial evolution, Machine learning

## Abstract

**Background:**

Tension pneumothorax is one of the leading causes of preventable death on the battlefield. Current prehospital diagnosis relies on a subjective clinical impression complemented by a manual thoracic and respiratory examination. These techniques are not fully applicable in field conditions and on the battlefield, where situational and environmental factors may impair clinical capabilities. We aimed to assemble a device able to sample, analyze, and classify the unique acoustic signatures of pneumothorax and hemothorax.

**Methods:**

Acoustic data was obtained with simultaneous use of two sensitive digital stethoscopes from the chest wall of an ex-vivo porcine model. Twelve second samples of acoustic data were obtained from the in-house assembled digital stethoscope system during mechanical ventilation. The thoracic cavity was injected with increasing volumes of 200, 400, 600, 800, and 1000 ml of air or saline to simulate pneumothorax and hemothorax, respectively. The data was analyzed using a multi-objective genetic algorithm that was used to develop an optimal mathematical detector through the process of artificial evolution, a cutting-edge approach in the artificial intelligence discipline.

**Results:**

The in-house assembled dual digital stethoscope system and developed genetic algorithm achieved an accuracy, sensitivity and specificity ranging from 64 to 100%, 63 to 100%, and 63 to 100%, respectively, in classifying acoustic signal as associated with pneumothorax or hemothorax at fluid injection levels of 400 ml or more, and regardless of background noise.

**Conclusions:**

We present a novel, objective device for rapid diagnosis of potentially lethal thoracic injuries. With further optimization, such a device could provide real-time detection and monitoring of pneumothorax and hemothorax in battlefield conditions.

**Supplementary Information:**

The online version contains supplementary material available at 10.1186/s40779-021-00319-2.

## Background

Traumatic pneumothorax, namely tension pneumothorax (tPTX) is one of the leading causes of preventable death on the battlefield [1–4]. These findings are consistent among researchers examining case fatality rates in various conflicts. tPTX was estimated to be the cause of up to 5% of preventable deaths among fatally wounded combat casualties in the Vietnam war [4]. Studies examining more recent conflicts exhibit similar findings, stating tPTX to be among the most common potentially survivable injuries [5–9]. tPTX may also be associated with hemothorax (HTX) [10, 11], which is independently associated with an increased risk for mortality [8]. Although tPTX may be seen in approximately 2% of patients who suffer from simple pneumothorax, it is more likely to appear in trauma settings in light of poor diagnosis and treatment means [12].

The pre-hospital diagnosis of pneumothorax (PTX) and HTX is determined following a point of injury physical examination. In patients with a small PTX, findings on physical examination may be indistinct from normal physical examination findings. While various imaging tests, such as computerized tomography and chest X-ray can confirm the diagnosis, they are not employed in the pre-hospital setting. Emergency ultrasonography, namely the Extended Focused Assessment with Sonography for Trauma (E-FAST) protocol, has been utilized by pre-hospital providers to evaluate for PTX in addition to HTX. Although favorable studies present high overall diagnostic accuracy for PTX detection in major trauma (> 97%) [13, 14], implementation of pre-hospital ultrasonography requires expensive equipment and advanced technical training for the clinician in ultrasonography, where are prohibitive for wide adoption of this technique in many pre-hospital settings.

**Fig. 1:**
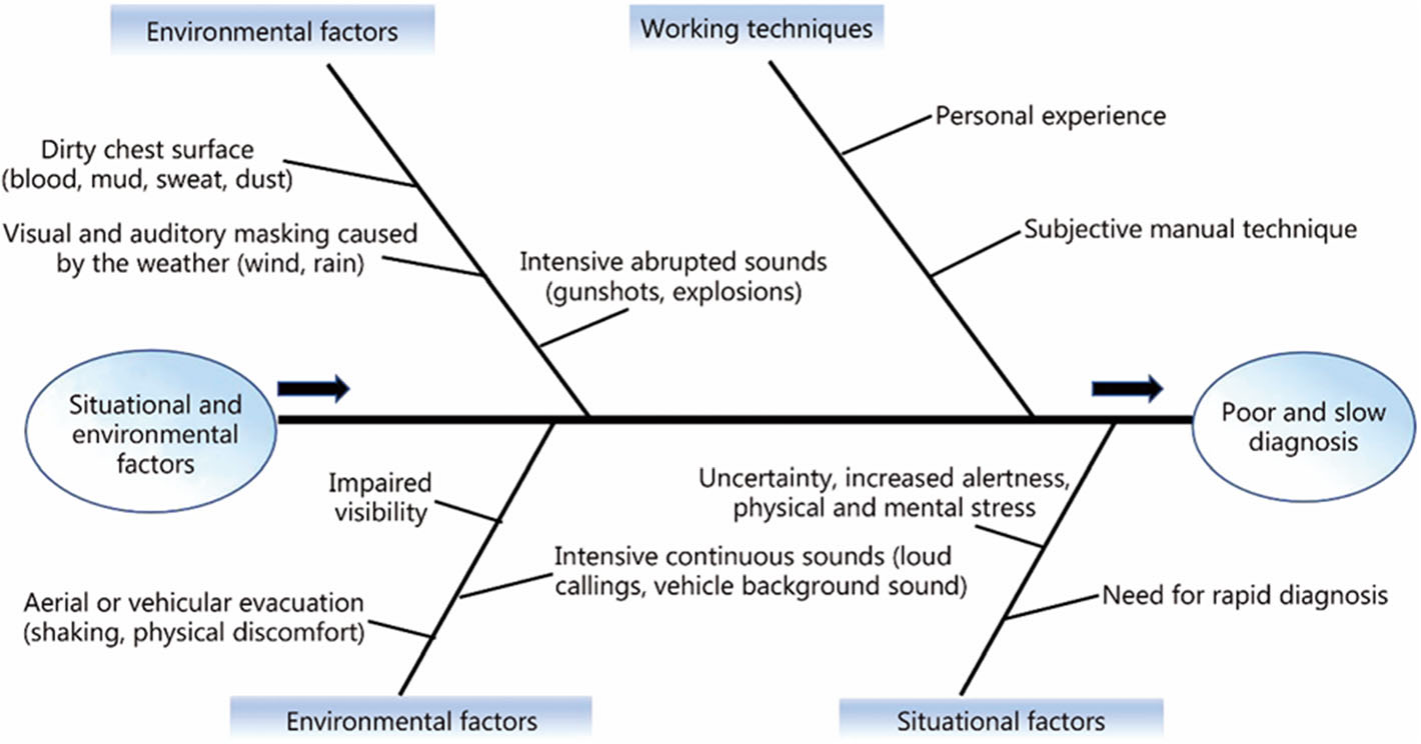
Environmental and situational cause and effect diagram

Accuracy of physical examination in patients who present with penetrating or blunt chest trauma varies as it is highly subjective and depends on the healthcare provider’s skills and experience. The sensitivity of auscultation was previously demonstrated to be as low as 50% in patients admitted with various degrees of hemopneumothorax due to penetrating or blunt chest trauma [15]. Likewise, Kong et al. [16] presented that standard physical examination, consisting of an overall clinical impression, respiratory rate, oxygen saturation, tracheal deviation, chest expansion, percussion and auscultation findings, in a major trauma center, exhibited low sensitivity in diagnosing PTX and HTX [59% (95% CI, 51–66)] and [79% (95% CI, 72–86)], respectively. Similar results for HTX, PTX, and various degrees of hemopneumothorax were also described by Chen et al. [17] especially in patients with gunshot wounds with auscultation missing HTX in volumes up to 600 ml, and hemopneumothorax in volumes up to 800 ml*.* Later, they demonstrated higher sensitivity rates, up to 84% in a prospective study conducted on 148 trauma patients [18]. As these studies were performed in a controlled quiet environment and under no time constraints, it is important to note that in field conditions, and specifically during combat, medical treatment is given under fire, in poor visibility, in the presence of intense background noise, under severe mental and physical stress, and under strict time limits. As such, physical examination may be extremely difficult to perform, and clinical impression may be obscured, leading to potential for decreased diagnostic accuracy as well as delays in diagnosis. Factors contributing to decreased performance in clinical diagnosis of tPTX and HTX in battlefield conditions are depicted in Fig. 1. There are currently several pre-hospital non-invasive technological solutions that may help diagnose PTX and HTX. These include ultrasound- [19–21], radio waves- [22–24], and infrared thermography-based techniques [25]. Nevertheless, these techniques do not provide a robust solution and present numerous limitations such as specifically low detection rate in obese or muscular patients [19], difficulty in identifying the injured side [26], and a wide range of overall accuracy rate [23, 24].

In this study, we present a prototype of a dual digital stethoscope sampling device which utilizes a novel algorithm with potential to non-invasively detect and quantify the presence of air and fluid in the plural cavity, independent of external factors that may delay diagnosis and treatment in pre-hospital battlefield conditions.

## Methods

### Study design

The study was conducted in four white domestic female pigs (Laboratory Animals Farm, Lahav, Israel), aged 4 months, (41–50) kg, housed in the institutional animal facility accredited by the Association for Assessment and Accreditation of Laboratory Animal Care International (AAALAC). Water and normal appropriate diet were available ad libitum. The experimental procedures were performed 7 days after acclimatization. Food was withheld from the night before the procedure. Animals were sedated with Xylazine (1 mg/kg, IM, Eurovet Animal Health BV, The Netherlands) and anesthesia was induced with Ketamine (10 mg/kg, IM, Vetoquinol SA, Lure Cedex, France). The ear vein was then cannulated for intravenous administration of a mixture of Diazepam (2 mg, IV, TEVA Pharmaceutical Industries Ltd., Noida, India), Ketamine (400 mg, IV), Propofol[(1–4) mg/kg, IV, Fresenius Kabi Austria Gmbh, Austria], and Tramadol (5 mg/kg, IM, Rafa Laboratories Ltd., Jerusalem, Israel) for analgesia. The pigs were then intubated with a cuffed silastic endotracheal tube (7.0 mm, Portex Tracheal Tube, UK). Anesthesia was maintained with 2% isoflurane (Piramal Critical Care Inc., PA) in 100% oxygen, and animals were ventilated using controlled mechanical ventilation (Excel 210-SE anesthesia machine Datex-Ohmeda Inc., Madison, WI) or Narkomed-2B Anesthesia Machine (North American Drager, PA). Tidal volume was set to 10 ml/kg with respiratory rate of 13 to 15 breaths per minute adjusted to an end-tidal CO_2_ of 35 mmHg at baseline. At the end of the protocol, the animals were euthanized with an intravenous injection of KCl solution (Fagron Group BV, Rotterdam, The Netherlands). The porcine model was chosen due to its physiological and anatomical similarity to the human thorax including chest wall thickness, subcutaneous fat distribution, skin thickness, intercostal spaces width, muscle tissue response, and lung positioning. Moreover, the volumes of porcine and humans’ lungs are considered comparable, with a total lung capacity of 55 ml/kg [27].

Twelve seconds recordings of breath sounds, representing three mechanical inhalation-exhalation cycles, were sampled over the right 7th intercostal space, at the mid-axillary line. This specific site was chosen based on previous studies performed by our group as an appropriate site for auscultation in similar-size animals. Preliminary baseline recordings were sampled and were defined as “volume 0”. After this, simple PTX was induced with increasing volumes of 200, 400, 600, 800, and 1000 ml of air, injected using a 100 ml syringe and a 14G needle into the pleural cavity and underneath the sampling site. Before simulating HTX, the thorax was emptied of air by using the same syringe, until air leak through the needle was absent. Then, an increasing volume of saline (0.9% sodium chloride) was injected in the same position as described above: 200, 400, 600, 800, and 1000 ml. Each of the aforementioned states was sampled with a twelve-second recording of breath sounds. The study was conducted in a quiet background, without restrictions on environmental noise during the recordings.

### Prototype assimilation

#### Apparatus assembly

**Fig. 2:**
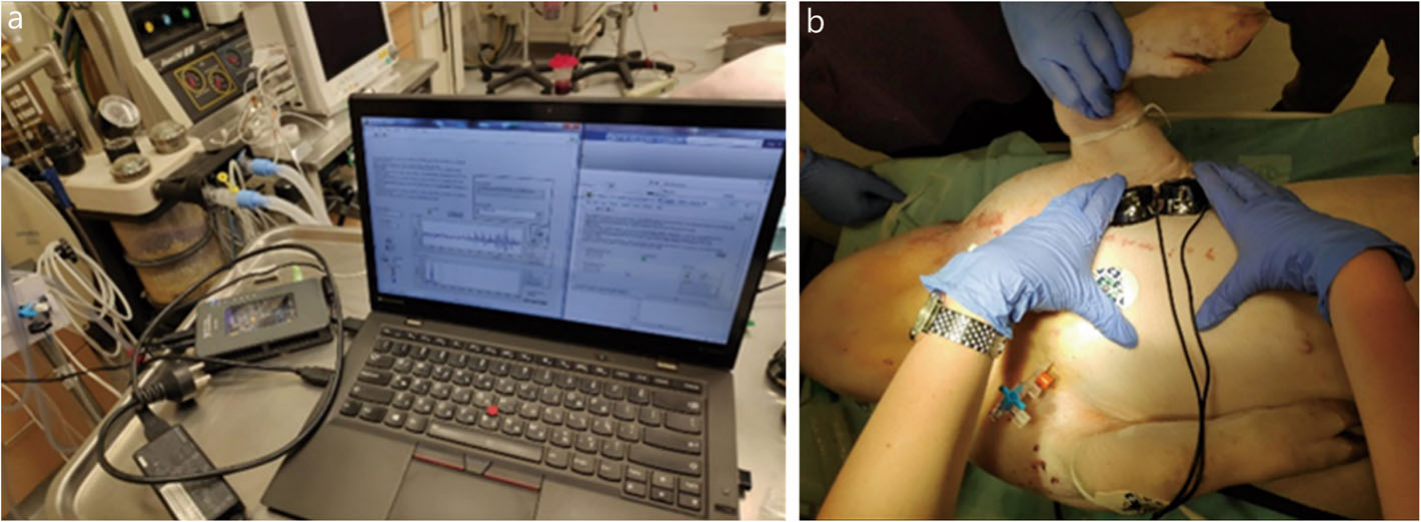
The sampling apparatus consisted of two Thinklabs One digital stethoscopes, connected to a portable myRIO-1900 embedded device. **a** A sampling card was connected on one side to the recording apparatus, and one the other end, to a personal computer running our dedicated algorithm. **b** The recording apparatus, consisting of two digital stethoscopes. Apparatus’ approximate dimensions: Length: 10 cm; Width: 5 cm; Height: 3 cm; Weight: 110 g

We chose to use two FDA- and CE-approved commercial Thinklabs One digital stethoscopes (Thinklabs Medical LLC, CO, USA) [28], capable of sampling a wide range of frequencies and amplifying them by up to 100-fold, allowing for recording over a thick chest wall if needed. The stethoscope is round-shaped, with a diameter of 46 mm and a height of 28 mm, it weighs about 50 g and is equipped with built-in filters that reduce background noise. The filter we chose was designed to enable a sampling of (20–2000) Hz +/− 3 dB. We placed two microphones next to each other, supported by a longitudinal sustaining frame. The microphones were connected to a spatial sampling system assembled at the IDF Medical Corps Medical Engineering Division. The system included a 3.5 mm adaptor connecting the stethoscopes to a portable myRIO-1900 embedded device (National Instruments, TX, USA) [29]. The sampling apparatus is presented in Fig. 2.

#### Software programming

The sampling algorithm was programmed by the IDF Medical Corps Medical Engineering Division using the LabVIEW platform. The samples recorded by the two stethoscopes were separated into two audio channels for each stethoscope. Therefore, for each recording made, four data sources were obtained. The recordings were analyzed using a MATLAB (Mathworks, Natick, MA, USA) computing environment and were filtered to omit high-pass and low-pass data points with frequencies of < 60 Hz and > 2000 Hz, respectively. For each recording, the intensity ($\frac{\mathrm{watt}}{\mathrm{m}2}$) of the signal was measured in constant gaps of 0.00005 s, i.e. 20,000 samples per second. In total, 176 datasets were obtained and were later extracted to an Excel sheet for further data processing and analysis by an external software house (Clinetix - Clinical Kinetics, Tel-Aviv, Israel). The working assumption was that each volume of simple PTX and HTX had a unique acoustic pattern that could be obtained and analyzed, and on which a classification algorithm could be programmed and enable the differentiation of the various volumes of injury compared to the normal state. Due to the small dataset, we decided not to use a model-based analytical method, such as an artificial neuronal network or a decision tree, which is a better fit for larger quantities of datasets. This led us to use a memory-based method, namely a k-nearest neighbors (KNN) algorithm, which generates a prediction by estimating how likely a data point is to be a member of one group or another, based on the distance between the data point and each group. Using the KNN algorithm’s parameters as input, a multi-objective genetic algorithm (MOGA) was utilized to develop a mathematical detector through the process of artificial evolution.

### Statistical analysis

Sensitivity, specificity, and accuracy for each injury volume were calculated. Accuracy was defined as $\frac{\mathrm{TP}+\mathrm{TN}}{\mathrm{TP}+\mathrm{TN}+\mathrm{FP}+\mathrm{FN}}$ (TP-True positive, TN-True negative, FP-False positive, FN-False negative). In addition to comparing each of the volumes separately from each other, we conducted additional testing combining all observations with comparison to baseline (“volume 0”). A Pearson correlation coefficient was computed to assess the relationship between the real value and the predicted one. Calculations were made using a C# programming language in a LINQPad software utility.

## Results

Each twelve-second recording consisted of 240,000 samples, with a total of 176 datasets obtained. A visual representation of the data was also produced as depicted in Additional files. The KNN algorithm consisted of a cross-validation process that separated the data into a single testing set and the remaining 175 training sets. Iteratively, we compared each testing set to the complementary training set for a total of 176 different iterations. The algorithm then calculated the number of most identical datasets (K) for each testing set. Of the K results returned, the majority decision determined the success or failure of the prediction. Preliminary analysis of the data found that the samples of 200 ml PTX and HTX were very similar in their acoustic properties to that of a normal state, and therefore we decided not to include them in future calculations, in order to improve the algorithm’s classification capability.

### PTX and/or HTX differentiation in comparison to baseline

The algorithm succeeded in predicting the presence of PTX and/or HTX at each of the different volumes combined in comparison to baseline, with 81% sensitivity, 63% specificity, and 80% accuracy. The term “combined” refers to the unification of any of the measured volumes of PTX and/or HTX. Thus, addressing it as a single condition without distinguishing between the types of injury.

### PTX differentiation

When the algorithm was tested to predict the presence of PTX at each of the various volumes combined, in comparison to baseline and/or in comparison to the presence of HTX, at each of the various volumes combined, we achieved 63% sensitivity, 94% specificity, and 80% accuracy.

We demonstrated that the algorithm can predict the presence of PTX at each of the various volumes combined, in comparison to baseline with 63% sensitivity, 69% specificity, and 64% accuracy. When we tested the ability of the algorithm to distinguish between the various volumes of PTX, in comparison to baseline, we achieved an average level of sensitivity, specificity, and accuracy of 80% each.

### HTX differentiation

Similar calculations in HTX at each of the various volumes combined, in comparison to baseline, achieved 100% sensitivity, 100% specificity, and 100% accuracy.

When the algorithm was tested to predict the presence of HTX at each of the various volumes combined vs. the presence of PTX at each of the various volumes combined, we achieved 100% sensitivity, 100% specificity, and 100% accuracy.

Statistically significant positive correlation was found between the real volumes and the predicted ones for PTX [*r* (72) = 0.23, *P* = 0.034], HTX [*r* (78) = 0.81, *P* < 0.0001], and PTX and HTX combined [*r* (142) = 0.90, *P* < 0.0001]. Table 1 illustrates each of the above-mentioned measurements.

**Table 1:** Sensitivity, specificity, and accuracy in the various conditions tested

Differentiation level (conditions tested)	Sensitivity (%)	Specificity (%)	Accuracy (%)	*r*	*P* value
Normal; PTX (exc. 200 ml) and / or HTX (exc. 200 ml)	81	63	80	0.90	< 0.0001
Normal; PTX (exc. 200 ml); HTX (exc. 200 ml)	63	94	80	0.90	< 0.0001
Normal; PTX (exc. 200 ml)	63	69	64	0.23	0.034
Normal; PTX 400 ml; PTX 600 ml; PTX 800 ml; PTX 1000 ml	80	80	80	–	–
Normal; HTX (inc. 200 ml)	100	100	100	0.81	< 0.0001
HTX (exc. 200 ml); PTX (exc. 200 ml)	100	100	100	0.81	< 0.0001

*PTX* pneumothorax, *HTX* hemothorax, *inc* including, *exc* excluding

## Discussion

Technological advances in recent years have resulted in the implementation of artificial intelligence (AI) applications in medicine in general, and in emergency medicine in particular [28–30]. In our study, we introduce an AI-based method capable of accurately identifying air and fluid accumulation in the pleural cavity and assessing their volumes. The algorithm was able to detect the accumulation of (400–1000) ml of air and/or fluid in the pleural cavity, and distinguish it from a normal state within twelve-seconds and regardless of background noise, with an accuracy ranging from 64 to 100%. The algorithm was able to detect the presence of PTX or HTX with a sensitivity of 81%, a specificity of 63% and an accuracy of 80%. When attempting to specifically identify PTX, the device achieved a lower sensitivity rate of 63%, but with higher specificity of 94% and overall accuracy of 80%. When we attempted to classify the exact volume of the injury, in the range of 400 ml to 1000 ml, accuracy dropped to 64%. The algorithm identified HTX with 100% sensitivity, 100% specificity, and 100% accuracy. However, the algorithm was not able to accurately predict distinct volumes of HTX. This may be due to a lack of training data for the algorithm, experimental design or intrinsic acoustic properties of HTX and further studies will be needed to elucidate this issue. Preliminary examination of the acoustic properties of the HTX samples exhibited a low signal-to-noise ratio, indicating a relatively high rate of background noise in comparison to the level of the desired signal. We believe this was due to the induction of HTX at the same physical location, shortly after draining out the air that was inserted earlier for the purpose of PTX induction. It is possible that some residual air remained in the pleural cavity, accentuating the presence of the injury beyond the increasing volume of saline. Nevertheless, it is important to note that we applied various computational methods, such as the Monte Carlo method, to evaluate the quality and the arbitrariness of the data and found it to be consistently balanced and protected from bias or overfitting effects. This means that the noise or random oscillations in the training set did not impair the prediction ability of the algorithm. This also means that given more data, it is likely that we will be able to significantly increase the prediction accuracy.

Although data describing morbidity and mortality of combat casualties exists from early conflicts in the twentieth century, most of the modern knowledge and experience are based on US military operations in the Middle East, specifically in Iraq and Afghanistan. It is important to note that in relation to earlier conflicts, modern conflicts are characterized by increased utilization of explosives, such as improvised explosive devices (IEDs), grenades, and shoulder-fired missiles, resulting in higher rates of blast injuries and concomitant blunt thoracic injuries which are difficult and less intuitive to diagnose [30]. A review performed by the Israel Defense Forces (IDF) Medical Corps after Operation Protective Edge in 2014, also suggested tPTX to be one of the most common causes of preventable death on the battlefield, alongside hemorrhage and airway compromise [31]. A population-based study examining PTX incidence in severe trauma patients in a civilian prehospital setting has shown that up to 20% of major trauma victims suffered from PTX with up to 15% of patients receiving emergency chest decompression [32]. Although considerable variation exists in the literature when addressing PTX size assessment, it is considered recommended to treat it when 30–50% of the hemithorax (600–1000) ml respectively, is affected [33–35]. Considering the low clinical significance of a 200 ml PTX, and due to the technical issues described above, we decided to exclude those samples from calculations. The samples were recorded in the presence of mild background noise, and although its level was not measured, it can be inferred from the literature as the mean noise level in operating rooms is considered to be around (51–75) dB, as described by Hasfeldt et al. [36] in their review. Due to the limitations of in vivo human experiments, the porcine model was chosen to demonstrate the feasibility, primarily due to its physiological and anatomical similarity to the human thorax. Previous studies described porcine pneumothorax models to be physiologically relevant to human extrapolation [27]. Future experiments should utilize the algorithm in human cadavers to construct human-based datasets.

A genetic algorithm was used to create the mathematical detector which identified the unique differentiating signature for each volume of PTX and/or HTX. Genetic algorithms are a family of optimization algorithms designed to streamline problem-solving by artificially activating a process that simulates the natural selection process underpinning the theory of evolution [37]. The method is based on applying small changes (“mutations”) to randomly selected solutions for a certain problem, while producing new solutions and selecting those that better fit the target that has been defined by a “fitness” function and its stopping criteria until reaching a near-perfect optimization within several runs (“generations”). This method is best used for solving problems for which the target is known but the way to attain it is unclear.

Among the contemporary technological solutions for prehospital thoracic trauma detection, the most widespread modality is emergency ultrasonography. The E-FAST protocol may assist in assessing the presence of PTX and HTX, and to a lesser extent, its severity. Although the majority of studies present a very high specificity (> 95%) for the diagnosis of PTX in patients with thoracic trauma, they also exhibit a low sensitivity (~ 50%) when compared to other imaging modalities [38]. Prehospital emergency ultrasonography is noninvasive, portable, and inexpensive. Moreover, it has the advantage of being functional even in loud surroundings.

Nevertheless, it is heavily dependent on provider experience and therefore may be time consuming and difficult to implement in settings with a need for a large number of simultaneously deployed pre-hospital healthcare providers in a warzone. Additionally, E-FAST cannot differentiate between an acute HTX and a chronic pleural effusion [39]. Although the latter may provide a challenge for our modality as well, we offer a non-provider-dependent rapid and objective detection modality, which may provide the actual volume of injury with higher sensitivity rate.

Diagnosing PTX using various acoustic techniques has previously been demonstrated in the literature. In their exploratory study, Mansy et al. [38] demonstrated how the presence of PTX affects the transmission of sound waves across the thorax. During the study, which was conducted on 19 patients with iatrogenic PTX. Although the authors suggest a potential method for distinguishing between baseline and the presence of PTX, they present an invasive method. Peng et al. [39] presented a computational model simulating acoustic transmission from the lung to the thoracic surface in a porcine model under PTX. They found that introducing sound waves via the endotracheal tube may be measured by the transmitted waves from the thorax by a laser Doppler vibrometer. Thus, presenting a potential method for detecting pulmonary abnormalities, including PTX. A canine model for distinguishing PTX from baseline using vibrational response on the thorax following an external acoustic stimulation introduced via the endotracheal tube was described by Royston et al. [40]. Early studies describing the acoustic characteristics of breath sound suggested PTX cause amplitude reduction, decrease in high-frequency components, and reduced amplitude variation [41]. Hayashi [42] introduced an external device that analyzed and converted the frequency of thoracic auscultatory sounds to numerical values and demonstrated how the presence of PTX could be detected with a sensitivity and specificity of 71.4 and 100%, respectively. His results were based on ten breath cycle recordings, which are equivalent to about (30–40) seconds. Confirmatory diagnosis of PTX was made by chest X-ray or a chest tomography and no data regarding the volume of the injury was provided [42].

In view of current literature, our method presents higher sensitivity and accuracy with shorter measurement times, regardless of background noise, and distinguishing PTX and HTX as small as 400 ml in volume. Moreover, our method utilized auscultatory data from a single spot on the thorax with no need for external excitation of any kind. Furthermore, while most studies refer to PTX diagnosis only, we managed to identify the presence of injected fluid in the pleural cavity, simulating HTX. Another clinical implication of our modality is that in the case of HTX, optimal resuscitation could be applied, and additional medication could be administered, such as tranexamic acid or fresh frozen plasma infusion. Moreover, chest tube positioning might be different if prior knowledge is available. In the case of PTX, one should point the tip of the tube upwards, while in the case of HTX, it should be pointed posteriorly and caudally. Due to rapid application of our modality, a near-instant follow-up could be applied, and patients treated with chest tubes could be monitored regardless of background noise. Equally important, proper use of our device can prevent unnecessary treatment in patients who do not require chest drainage.

For PTX and HTX simulation, we induced an isolated injury while in the real-world, there may be a combined injury resulting in hemopneumothorax and concomitant pulmonary contusions that may potentially affect the results of auscultation. Moreover, natural breathing was absent due to the ventilated animal model and the absence of normal blood flow and pulse may also have influenced the acoustic properties of the respiratory sounds. Another limitation to this study was the lack of measurement of background noise. Thus, it is possible that the background noise may have caused artifacts in the signals detected. Nevertheless, the stethoscopes were designed to pick up signals coming from directly in front of them only; so that the potential interruption of background noise was minimal if existing at all. In addition, we applied two levels of filtering, the first was the built-in filter in the stethoscope and the second was the frequency filter we built as part of the sampling algorithm. As our modality is designated to be utilized in field conditions, and on the battlefield, future studied would address that point and test our algorithm’s ability to sample and classify the signals even when applied in loud environments, of up to 120 dB. Next, although we sampled a high number of measurements per second, the study comprised of only four animal models. Thus, selection bias and overfitting in the prediction algorithm should be taken into consideration. Moreover, the evaluation process was conducted through cross-validation. Nevertheless, several preliminary tests were performed to ensure the quality of the data despite the small amount of data. A Monte Carlo simulation method for estimating the arbitrariness of the correlations detected in the recorded samples showed that the recorded data was balanced, not arbitrary, and protected from bias or overfitting effects. No definitive measures, such as chest X-ray or thoracoscopy were used to confirm the location of the PTX/HTX. Therefore, we cannot say with absolute certainty whether the signal measurement was performed precisely above the injury. However, in real-world settings, auscultation will be done over an arbitrary spot on the patient’s hemithorax with no prior knowledge of the location of the injury, and we expect a diagnostic device could indicate the presence of PTX or HTX even if not placed directly above them.

## Conclusions

We have managed to detect and classify an abnormal accumulation of air and fluid in the pleural cavity in a porcine model simulating hemopneumothorax by utilizing a prediction algorithm which was optimized by an artificial evolution method. This novel method has the potential to non-invasively detect and quantify within seconds the presence of hemopneumothorax, independently of external factors that may delay diagnosis and treatment. Although promising, it seems that our modality can better distinguish fluid accumulation rather than air. Beyond the potential use in field conditions and on the battlefield, our method may also present potential benefits for use in trauma bays, intensive care units, catheterization rooms, and operating rooms. Further studies are required to improve and better assess the algorithm’s accuracy and to design portable prototype.

## Supplementary Information

**Additional file 1: Table S1.** A tabular time-series representation of the intensity measured at each volume across time.

**Additional file 2: Figure S1**. A visual illustration of the filtered time-series. Horizontal axis: time series; Vertical axis: intensity.

## Data Availability

The datasets generated and/or analyzed during the current study are not publicly available due to intellectual property protection but are available from the corresponding author on reasonable request.

## Abbreviations

AI: Artificial intelligence
E-FAST: Extended focused assessment with sonography for trauma
HTX: Hemothorax
IEDs: Improvised explosive devices
KNN: K-nearest neighbors
MOGA: Multi-objective genetic algorithm
PTX: Pneumothorax
tPTX: Tension pneumothorax
