# Supplementary material for: Hemopneumothorax detection through the process of artificial evolution - a feasibility study
Source: Mil Med Res. 2021 Apr 25;8:27. doi: 10.1186/s40779-021-00319-2 (PMC8070275; doi:10.1186/s40779-021-00319-2)
Supplement: Supplementary file 1 — Additional file 1: Table S1. A tabular time-series representation of the intensity measured at each volume across time. [file 40779_2021_319_MOESM1_ESM.docx]

**Additional Table.** A tabular time-series representation of the intensity measured at each volume across time

| **Volume = 0** | **Volume = 200** | **Volume = 400** | **Volume = 600** | **Volume = 800** | **Volume = 1000** | **Time** |
| --- | --- | --- | --- | --- | --- | --- |
| -0.003147765 | 0.00527999 | -0.005280791 | 0.001282211 | 0.004940968 | 0.000057328 | 0.00005 |
| -0.003868620 | 0.010674365 | -0.00546393 | -0.003228215 | 0.008912077 | 0.000057328 | 0.00010 |
| -0.004535408 | 0.015915245 | -0.005574587 | -0.007687208 | 0.01277273 | 0.000057328 | 0.00015 |
| -0.005138628 | 0.020934398 | -0.005609785 | -0.012037042 | 0.016472957 | 0.000057328 | 0.00020 |
| -0.005669726 | 0.025666969 | -0.005567618 | -0.016221648 | 0.019965008 | 0.000057328 | 0.00025 |
| -0.006121227 | 0.030052391 | -0.005447304 | -0.020187422 | 0.023204024 | 0.000057328 | 0.00030 |
| -0.006486846 | 0.034035231 | -0.005249207 | -0.02388397 | 0.026148677 | 0.000057328 | 0.00035 |
| -0.006761593 | 0.037565961 | -0.004974859 | -0.027264828 | 0.028761736 | 0.000057328 | 0.00040 |
| -0.006941843 | 0.040601624 | -0.004626945 | -0.030288097 | 0.031010593 | 0.000057328 | 0.00045 |
| -0.007025390 | 0.043106403 | -0.004209288 | -0.032917023 | 0.032867704 | 0.000057328 | 0.00050 |
| -0.007011484 | 0.045052081 | -0.003726804 | -0.035120491 | 0.034310955 | 0.000057328 | 0.00055 |
| -0.006900833 | 0.046418382 | -0.003185438 | -0.036873436 | 0.035323962 | 0.000057328 | 0.00060 |
| -0.006695596 | 0.047193181 | -0.0025921 | -0.038157155 | 0.035896261 | 0.000057328 | 0.00065 |
| -0.006399338 | 0.047372607 | -0.00195456 | -0.038959528 | 0.036023429 | 0.000057328 | 0.00070 |
| -0.006016975 | 0.046961008 | -0.001281348 | -0.039275135 | 0.035707111 | 0.000057328 | 0.00075 |
| -0.005554690 | 0.045970797 | -0.000581641 | -0.039105278 | 0.03495495 | 0.000057328 | 0.00080 |
| -0.005019834 | 0.04442218 | 0.000134881 | -0.038457894 | 0.033780437 | 0.000057328 | 0.00085 |
| -0.004420803 | 0.042342775 | 0.000858163 | -0.037347371 | 0.032202676 | 0.000057328 | 0.00090 |
| -0.003766907 | 0.03976711 | 0.001577928 | -0.035794276 | 0.03024606 | 0.000057328 | 0.00095 |
| -0.003068216 | 0.036736034 | 0.002283829 | -0.033824991 | 0.027939881 | 0.000057328 | 0.0010 |
| -0.002335400 | 0.033296046 | 0.0029656 | -0.031471256 | 0.02531787 | 0.000057328 | 0.00105 |
| -0.001579559 | 0.029498537 | 0.003613215 | -0.028769647 | 0.022417662 | 0.000057328 | 0.00110 |
| -0.000812048 | 0.025398972 | 0.004217039 | -0.025760982 | 0.019280232 | 0.000057328 | 0.00115 |
