# Supplementary figures and images for: Hemopneumothorax detection through the process of artificial evolution - a feasibility study
Source: Mil Med Res. 2021 Apr 25;8:27. doi: 10.1186/s40779-021-00319-2 (PMC8070275; doi:10.1186/s40779-021-00319-2)

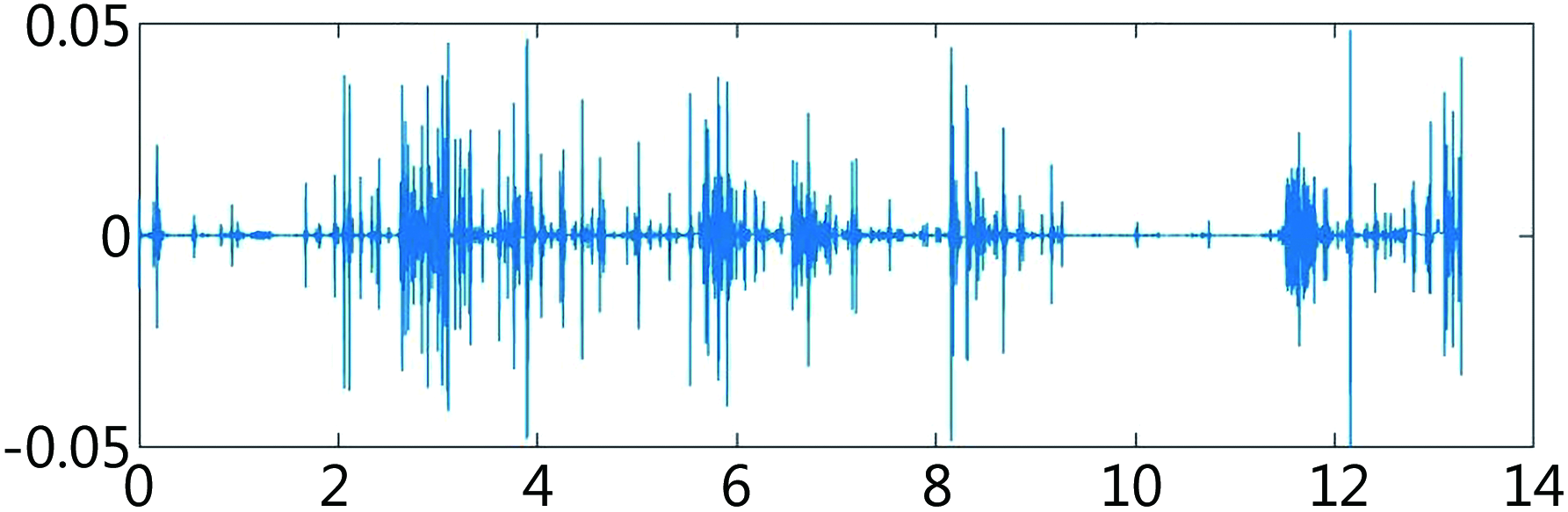

Supplement: Supplementary file 2 — Additional file 2: Figure S1. A visual illustration of the filtered time-series. Horizontal axis: time series; Vertical axis: intensity. [file 40779_2021_319_MOESM2_ESM.tif]
